# Combined Bile Duct and Pancreatic Duct Injuries during Distal Gastrectomy for Obstructing Peptic Ulcer Disease

**DOI:** 10.1155/2020/8821601

**Published:** 2020-10-15

**Authors:** Narendra Pandit, Kunal Bikram Deo

**Affiliations:** Division of Surgical Gastroenterology, Department of Surgery, B P Koirala Institute of Health Sciences (BPKIHS), Dharan, Nepal

## Abstract

A combination of bile and pancreatic duct injuries is very rare. Anomalous ductal anatomy, distorting duodenal fibrosis, and pancreatic atrophy predispose to this untoward complication during performance of distal gastrectomy for benign peptic stricture. The technical challenges posed by this complication and experience gained by managing it are shared.

## 1. Introduction

An easy access to potent medication and endoscopic facilities has rendered peptic pyloric strictures (PPS) rather uncommon. However, once formed, the resultant gastric outlet obstruction (GOO) often necessitates surgical intervention. Truncal vagotomy and antrectomy remain the gold standard in its treatment [1]. The intensity of fibrosis and the resultant distortion of anatomy are known to predispose the subjacent bile duct injury and in rare cases the pancreatic duct as well [2]. Intraoperative detection of such injuries could be abstruse and their treatment exigent. We report the challenges posed by such a case and discuss the management strategy for a successful outcome.

## 2. Case Report

**Figure 1:**
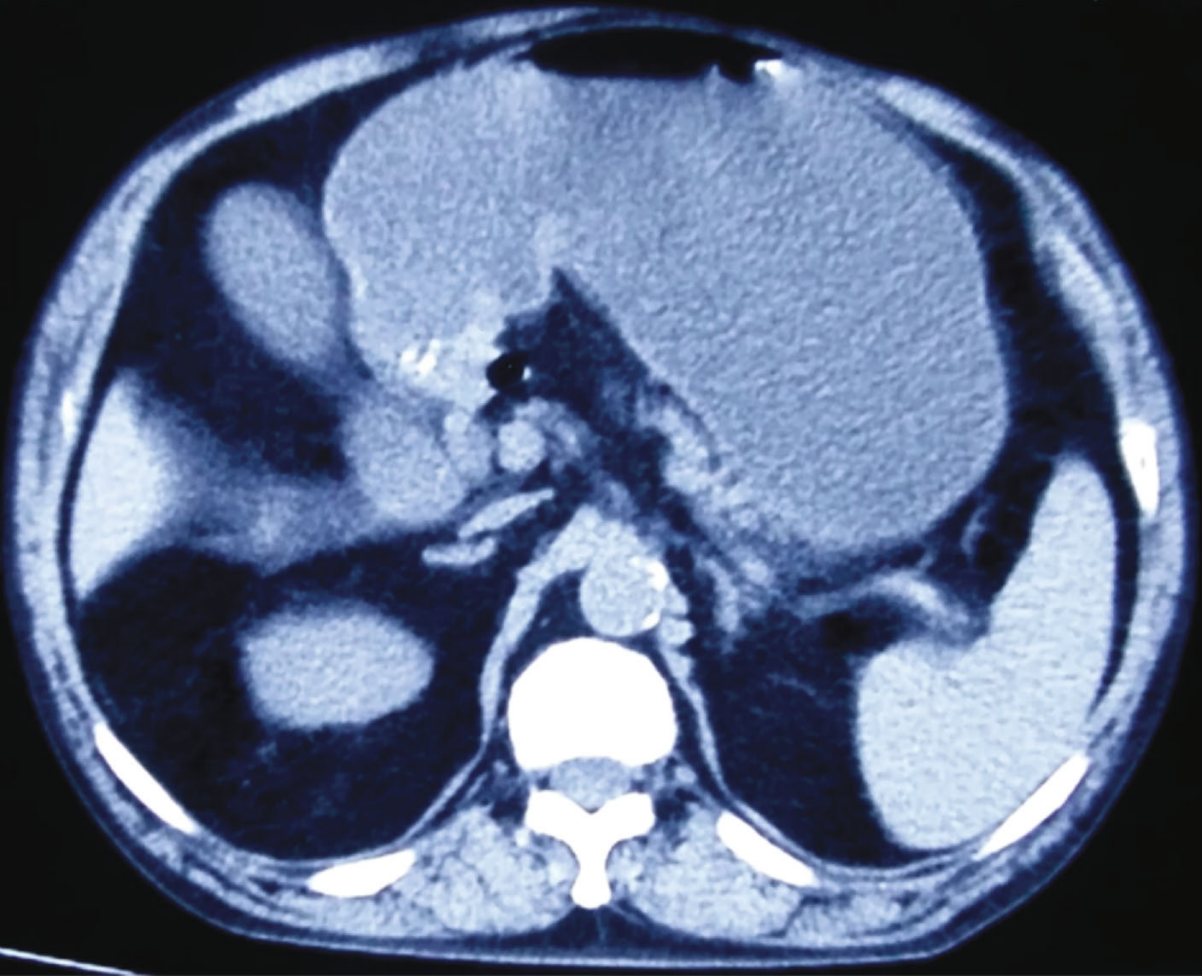
CT image showing distended stomach, concentric thickened pylorus without mass lesion, and an atrophic pancreas.

**Figure 2:**
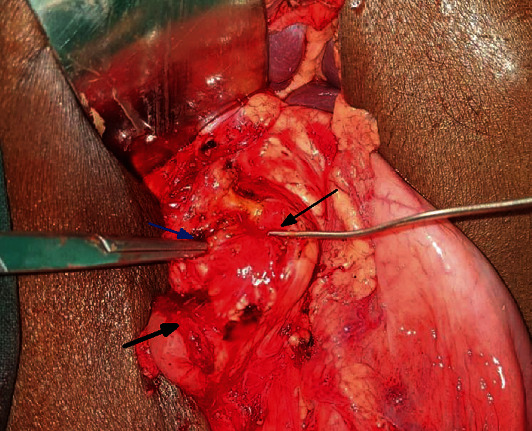
Intraoperative view showing combined bile duct (*probed and black arrow*) and pancreatic duct (*probed*, *blue arrow*) injuries. Note that the bile stained field and the divided first part of the duodenum (*black arrow*).

A 45-year-old male is presented with recurrent epigastric pain and vomiting of 2 years duration. The vomitus was large volume, nonbilious, and containing semidigested food. There was no history of significant weight loss or gastrointestinal bleed. Examination was unremarkable other than mild dehydration and a distended stomach with succussion splash. A nasogastric tube drained 1.2 liters of gastric content. Endoscopy revealed a grossly dilated stomach with an impassable pyloric stenosis but no mass lesion. Computed tomography (CT) abdomen concurred with endoscopy and biopsy confirmed absence of malignancy (Figure 1). With a diagnosis of peptic pyloric stricture, the patient was optimized and taken up for antrectomy and truncal vagotomy. There was an intense fibrosis distorting pyloro-duodenum, which on dividing extruded bile. Further examination and probing revealed an inadvertent division of both bile and pancreatic ducts (Figure 2). The pancreatic head was atrophic, and there was an anomalous union of pancreatic duct on bile duct (P-B type). In view of the above, pancreatoduodenectomy (PD) was performed, which took 270 minutes and entailed a blood loss of 280 ml. Postoperative periods were uneventful, and he was discharged on day 12. The histopathological report confirmed benign peptic stricture, and the patient is doing well at 16 months follow-up.

## 3. Discussion

Iatrogenic dual ductal injuries are not only uncommon but are also probably underreported [3]. A combination of anomalous ductal anatomy, distorting duodenal fibrosis, and pancreatic atrophy predisposed to this untoward complication [4]. The above issues also render the detection of these injuries exigent; hence, awareness of such a complication is necessary. Wide exposure, adequate Kocherization of the duodenum, and probing are necessary to assess the extent of damage. Intraoperative cholangiogram/ductogram might additionally help.

Although reimplantation of ducts into the duodenum could be attempted over a stent, but atrophic pancreatic head, fear of leak/late stricture, and subsequent complications rendered this option undesirable. In addition, Roux-en-Y hepaticojejunostomy for injured bile duct along with implanting the injured pancreatic duct into the sidewall of the duodenum after primary end-to-end duct anastomosis with PDS 6/0 interrupted over 4Fr double J stent (after cutting one end), though technically demanding offers other option [5]. Alternatively, transected pancreatic duct can also be dealt with Beger procedure or pancreaticogastrostomy.

Whilst nondilated bile/pancreatic duct and soft pancreas are factors that predispose to leak, but our expertise with PD, being high volume centre with low morbidity and mortality, tilted our decision towards electing this procedure for salvage [6, 7]. Soft pancreas with narrow ducts is better dunked into the stomach due to the technical ease. However, at expert hands with the aid of intraoperative loupe, duct-to-mucosa pancreatojejunostomy (modified Heidelberg technique) is an alternative with excellent outcome as was performed in the present case [8]. The nondilated bile duct (for hepaticojejunostomy) is better dealt with by wide spatulation or by using a Miller's cuff at the level of cystic duct insertion for narrowing free anastomosis to the jejunum.

We were proved right in our choice of salvage procedure as evident from an uneventful recovery and morbidity free life of the patient on 16 months follow-up.

## 4. Conclusions

Iatrogenic injuries of pancreatic and bile duct are uncommon but possible during antrectomy for benign pyloric stenosis. Awareness of this complication is mandatory for prompt intraoperative detection. Pancreatoduodenectomy proves efficacious in addressing this complication.

## Data Availability

None to reveal.
